# Rhabdomyolysis following status epilepticus with hyperuricemia: A case report and literature review

**DOI:** 10.1097/MD.0000000000011281

**Published:** 2018-06-29

**Authors:** Lingxing Wang, Shanyan Hong, Honghong Huang, Meili Yang

**Affiliations:** Department of Neurology, Second Affiliated Hospital of Fujian Medical University, Quanzhou, Fujian, China.

**Keywords:** hyperuricemia, rhabdomyolysis, status epilepticus

## Abstract

**Rationale:**

Rhabdomyolysis owing to status epilepticus (SE) can be life-threating, with acute kidney injury (AKI) the most serious complication; therefore, early recognition of the risk factors is important. Hyperuricemia after epileptic seizures has been reported, and severe hyperuricemia can lead to acute renal function damage.

**Patient concerns:**

We present the case of a 21-year-old man hospitalized for SE, who had especially high level of blood uric acid (UA) at initial presentation.

**Diagnosis:**

The patient was diagnosed with rhabdomyolysis due to SE.

**Interventions:**

The patient was treated with hydration and bicarbonate therapy. But he developed acute kidney failure (AKF) and hemodialysis was performed.

**Outcomes:**

After hemodialysis, his symptoms disappeared and laboratory data returned to normal.

**Lessons:**

Hyperuricemia after SE might indicate severe muscle damage or reduced clearance of metabolites, and could be a risk factor for kidney dysfunction, especially with rhabdomyolysis. To our knowledge, this is the first report of rhabdomyolysis following SE with hyperuricemia.

## 1 Introduction

SE is defined as epileptic seizure lasting more than 5 minutes or recurrent seizure episodes within 5 minutes without returning to preconvulsive neurological baseline.^[1]^ SE can produce rapid skeletal muscle break down and result in life-threating rhabdomyolysis. Other causes of rhabdomyolysis are muscle trauma, heat stroke, infection, and ingestion of certain drugs or toxins. There are no clear data on the morbidity of rhabdomyolysis in SE and there have been limited case reports in the past 30 years. Acute renal injury, which is the most serious complication of rhabdomyolysis, occurs in about 15% patients with rhabdomyolysis.^[2]^ Hyperuricemia after epilepsy has been reported, and UA is considered an effective antioxidant that can counteract oxidative stress during epileptic seizure.^[3]^ However, severe hyperuricemia might lead to acute renal function damage. There are no reports of rhabdomyolysis following SE with hyperuricemia. Here we report a case of rhabdomyolysis resulting from SE, which had surprisingly high levels of blood UA in the very early period. Medical Research Ethics Committee of Second Hospital Affiliated to Fujian Medical University approved this study. Written informed consent was obtained form the patient for publication.

## 2 Case report

A 21-year-old male was admitted to the emergency room in our hospital for generalized tonic–clonic seizures. Two hours before presentation, he had at least 7 or 8 episodes and did not recover consciousness between episodes. The patient had a 5-year history of epilepsy. He had stopped taking antiepileptic medication 4 years ago and experienced a generalized tonic–clonic seizure every 2 to 3 months. There was no history of trauma, fever, illicit drug use, or alcohol abuse. On presentation, the patient's temperature was 37.8°C, heart rate was 118/min and his blood pressure was 95/60 mm Hg. He was in coma and no focal neurological deficit was observed. Intravenous 10 mg diazepam was administered immediately and no more seizures were observed. Sodium valproate 0.4 g 3 times a day was administered via a feeding tube. Laboratory test results are shown in Table 1. Initial levels of blood UA were very high (989 μmol/L), and the patient also had increased creatinine (177 μmol/L), sodium (159.5 mmol/L), and magnesium levels (1.48 mmol/L). Brain computed tomography (CT) showed no lesion in the brain or chest. Ultrasound scanning of the kidneys was normal.

**Table 1:**
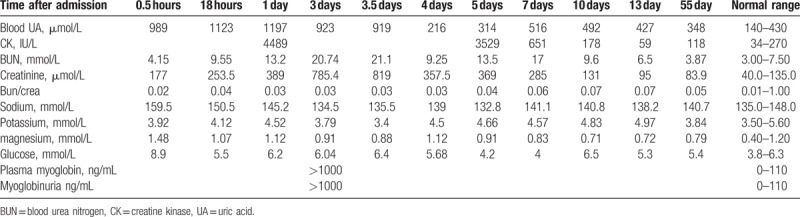
Laboratory data of the patient.

Fifteen hours after presentation, the patient was conscious and began to complain of back pain, although there was no bruising or trauma. The level of blood UA, blood urea nitrogen (BUN) and creatinine increased to 1123 μmol/L, 9.55 mmol/L, and 253.5 μmol/L, respectively. His blood pressure was 105/60 mm Hg, and he was treated with hydration and bicarbonate therapy. One day after admission, the level of creatine kinase (CK) was 4489 IU/L and blood UA (1197 μmol/L), BUN (13.2 mmol/L), and creatinine levels (389 μmol/L) had increased to higher levels. There was no decreased urine output and hydration with bicarbonate therapy was continued. Three days after admission, reduced urine output was observed although the urine was clear, and blood creatinine was elevated at 785.4 μmol/L. Plasma myoglobin was increased and myoglobinuria was present. Urine was positive for blood but negative for erythrocytes. The patient was diagnosed with acute renal failure owing to rhabdomyolysis following SE, and hemodialysis was performed. The patient improved and laboratory parameters trended to declined. He was discharged and recovered fully after 2 weeks in the hospital. His renal function, blood UA, and CK were in normal level after 6 weeks of hospital discharge.

## 3 Discussion

In the past 8 years, about 152 patients have been diagnosed with SE and have been admitted to our department. Among these, only 3 patients were diagnosed with rhabdomyolysis owing to SE and only one in the 3 patients developed acute kidney failure (AKF). Here we choose this case to report. In this case, the clinical features of obviously increased CK level and high levels of serum or urine myoglobin are consistent with the criteria of rhabdomyolysis.^[4]^ During SE, muscles may be severely injured and large amounts of muscle cell contents are released into circulation, which is known as rhabdomyolysis. There has been speculation that the stronger the epileptic convulsions, the more severe the muscle damage and the greater the likelihood that rhabdomyolysis will occur. There have also been reports of a single seizure leading to rhabdomyolysis.^[5]^ Therefore, it seems that the number and duration of seizures are related to the incidence of rhabdomyolysis; however, these are not the only risk factors.

An early clinical indicator for rhabdomyolysis owing to SE is muscle weakness or pain. However, after convulsive epilepsy, many patients might have these symptoms, so they are nonspecific and can be easily ignored. Another clinical sign is dark brown urine. But our patient did not have urine color change. Moreover, he had AKF and required hemodialysis. In normal physiology, plasma myoglobin, which is a dark red protein, binds with haptoglobin; the serum concentrations of myoglobin are very low in circulation. During rhabdomyolysis, the breakdown of muscle cells causes the release of large amounts of myoglobin into circulation, resulting in high serum myoglobin concentrations. Although myoglobin has a short half-life, we still found that in our case, serum levels of myoglobin were far above the upper limit of normal, indicating severe muscle damage. When the plasma level of myoglobin is higher than 0.5–1.5 mg/dL, it exceeds the haptoglobin binding capacity and myoglobin is excreted in the urine, which is termed myoglobinuria.^[6]^ However, only when the plasma concentration of myoglobin reaches 100 mg/dL, which corresponds to the destruction of 200 g of muscle tissue, the level of myoglobin in the urine is sufficient to be visibly tea- or cola-colored.^[7]^ Therefore, colored urine is not necessarily always present in rhabdomyolysis, which may explain the clear urine in our case. When myoglobin is filtered by glomeruli, it might form casts and obstruct the tubules, which finally results in acute renal failure.^[5]^ Obstructed tubules might prevent the excretion of myoglobin into the urine, leading to clear urine together with elevated plasma myoglobin. This might be another explanation for the clear-colored urine in this case. Thus, brown or dark brown urine is not a sensitive indicator of rhabdomyolysis after SE.

Urine test results that are positive for blood, but without red blood cells, might be helpful for the detection of rhabdomyolysis, given that there is 80% sensitivity.^[8]^ The most reliable and sensitive clue for rhabdomyolysis is elevated CK level,^[9]^ which results from damaged muscle cells and peaks at 3 days after the last seizure.^[10]^ When the CK level is 5 times the upper limit of normal values, the diagnosis of rhabdomyolysis can be confirmed.^[2]^ When CK >5000 IU/L, the risk of renal damage is increased,^[11]^ but does not definitely occur unless there are other risk factors. In this case, CK was <5000 IU/L but the patient had obvious kidney damage. Although CK was detected on the first day after admission—which was not the peak time point—other comorbidities might have contributed to renal failure.

It is noteworthy that the initial level of blood UA was remarkably high in this patient. Blood UA is the end product of purine metabolism and most of it is excreted via urine. Elevated blood UA levels might be caused by increased production or reduced clearance of UA in the blood. In rhabdomyolysis after SE, massively damaged muscle cells might release endogenous purine, which is metabolized into UA, in a manner similar to the occurrence of hyperuricemia owing to massive tissue necrosis in cancer patients after administration of chemotherapy.^[12]^ In our case, hyperuricemia was detected in the initial blood test after only 2 hours of convulsive SE; however, no previous reports have explained whether epilepticus would cause the level of blood UA to increase so rapidly and to such a large magnitude.

Another explanation is the reduced clearance of blood UA. Dehydration is a common complication of SE, which can depress renal plasma flow and reduce the renal excretion of UA, finally resulting in hyperuricemia. But in this case, the level of UA does not decrease after hydration so it was difficult to define the role of dehydration played. Although this patient had increased blood creatinine, indicating kidney dysfunction, the high level of UA occurred together with normal BUN at the initial blood test; therefore, hyperuricemia secondary to renal failure is insufficient to explain the underlying mechanism. Conversely, hyperuricemia might exacerbate kidney dysfunction. UA is filtered by glomeruli and is nearly completely reabsorbed in the S1 segment of the proximal tubule.^[13]^ Tubular secretion follows, and reabsorption of most UA occurs again within the S2 segment of the proximal tubule, such that only a small amount of UA appears in the urine.^[13]^ In hyperuricemia, filtered UA might be further concentrated and precipitated in the tubules, which might exacerbate the obstruction caused by myoglobin. The intratubular deposition of UA crystals and myoglobin increase tubular pressure and decrease renal blood flow, which then slows glomerular filtration and results in renal dysfunction. We speculate that this might be the underlying mechanism of AKI in this case, and it also indicates that hyperuricemia after SE might exacerbate the risk of renal failure in rhabdomyolysis. With early detection, this might be avoidable.

## 4 Conclusions

In the case presented here, the exact role of hyperuricemia is unknown. At minimum, hyperuricemia indicates severe muscle injury, decreased clearance of metabolites, and might be a risk factor for kidney dysfunction after rhabdomyolysis resulting from SE. Hydration and urinary alkalinization are helpful to reduce levels of blood UA and prevent exacerbation of impaired kidney function.

## Author contributions

**Conceptualization:** Honghong Huang.

**Data curation:** Shanyan Hong.

**Investigation:** Honghong Huang.

**Writing – original draft:** lingxing wang.

**Writing – review & editing:** Meili Yang.
